# Nidogen-1 could play a role in diabetic kidney disease development in type 2 diabetes: a genome-wide association meta-analysis

**DOI:** 10.1186/s40246-022-00422-y

**Published:** 2022-10-21

**Authors:** Ahmed Khattab, Ali Torkamani

**Affiliations:** 1 Integrative Structural and Computational Biology, Scripps Research, 3344 North Torrey Pines Court, Suite 300, La Jolla, CA 92037 USA; 2 Scripps Research Translational Institute, La Jolla, CA 92037 USA

## Abstract

**Background:**

Diabetic kidney disease (DKD) affects about 40% of patients with diabetes. It is incurable and usually leads to end-stage renal disease (ESRD). The pathogenesis of DKD is still not fully understood, and the genetics of DKD have not yet been extensively studied. In this study, we investigate the genetic basis of DKD in type 2 diabetes (T2D) to provide more insights into the pathogenesis of the disease.

**Results:**

Using the data provided by the UK Biobank (UKBB), we performed a DKD genome-wide association study (GWAS) in 13,123 individuals with T2D as well as two creatinine estimated glomerular filtration rate (eGFR) GWA studies: one in 26,786 individuals with T2D and the other in 339,080 non-diabetic individuals. We also conducted a DKD GWAS meta-analysis combining our results with those published by the surrogate markers for micro- and macro-vascular hard endpoints for Innovative diabetes Tools (SUMMIT) consortium. We confirm two loci previously reported to be associated with chronic kidney disease (CKD) and eGFR in T2D. The UMOD-PDILT locus is associated with DKD (*P* = 1.17E−09) as well as creatinine eGFR in both people with T2D (*P* = 1.31E−15) and people without diabetes (*P* = 3.95E−73). The PRKAG2 locus is associated with creatinine eGFR in people with (*P* = 2.78E−10) and without (*P* = 5.65E−72) T2D. Our meta-analysis reveals a novel association between DKD and variant rs72763500 (chr1:236116561) which is a splicing quantitative trait locus (sQTL) for nidogen-1 (NID1) gene.

**Conclusion:**

Our data confirm two loci previously reported in association with CKD and creatinine eGFR in T2D. It also suggests that NID1, a major component of the renal tubular basement membrane, could play a role in DKD development in T2D. While our NID1 finding remains to be replicated, it is a step toward a more comprehensive understanding of DKD pathogenesis.

**Supplementary Information:**

The online version contains supplementary material available at 10.1186/s40246-022-00422-y.

## Background

Diabetes causes CKD in about 40% of patients with diabetes in the USA and is the leading cause of ESRD [1]. While DKD usually occurs after 10 years in type 1 diabetes (T1D), it can manifest at the time of diagnosis in T2D [2]. DKD is characterized by glomerular hyperfiltration at early stages and then progress to albuminuria, reduced eGFR, and subsequent ESRD [3]. These functional changes are accompanied by structural changes which include thickening of the glomerular basement membrane as well as capillary and tubular basement membranes [4].

Not all patients with diabetes develop DKD, and early heritability studies showed that DKD happens in family clusters [5, 6] which indicates genetic susceptibility. While the heritability of DKD is estimated to be 35% in T1D [7], it is only 8% in T2D [8] which could be due to the highly heterogeneous phenotype in T2D [9]. Although the genetics of T2D have been intensively explored by many GWA studies, the genetics of its complications have not received as much attention from researchers. In particular, the genetic of DKD is much less studied in T2D compared to DKD in T1D and most performed studies are of small sample sizes.

In 2003, the very first DKD GWAS in T2D was conducted using gene-based single nucleotide polymorphisms (SNPs) [10]. It initially identified the SLC12A3 gene as a candidate gene for DKD in a group of 188 (94 cases and 94 controls) Japanese individuals. The results were replicated in a larger group of 732 individuals (466 cases and 266 controls). In 2005, another GWAS using gene-based SNPs identified ELMO1 as a DKD-associated gene in a discovery cohort of 179 (87 cases and 92 controls) and a replication group of 701 (459 cases and 242 controls) Japanese individuals [11].

In 2009, a larger GWAS including 359,193 autosomal SNPs was performed in 820 cases and 885 controls of people with T1D identified four loci associated with DKD near FRMD3, CHN2-CPVL, CARS, and MYO16-IRS2 genes [12, 13]. These findings were replicated in African-American (965 cases and 1029 controls) [14], European (743 cases and 646 controls) [15], and in Japanese [16] cohorts with T2D.

In 2018, the SUMMIT consortium conducted the largest DKD GWAS [8, 17]. The study included seven phenotypes and a total of 26,827 individuals with T2D (12,710 with DKD). The main DKD phenotype included 5,717 individuals (3,345 DKD cases and 2,372 controls) with T2D. The GWAS identified a novel variant near GABRR1 gene associated with microalbuminuria in T2D.

One of the core advantages of conducting GWA studies is gaining insights into the pathophysiology of human diseases. Despite the effort to uncover the genetic basis of DKD, the heterogeneous phenotype and the complexity of the disease made the GWA studies challenging and limited their replicability [9, 18]. Up to now, these factors have restricted our full understanding of the pathogenesis of DKD.

Here, we conducted a GWAS for DKD in Europeans with T2D using data from the UKBB. To further explore the results of the DKD GWAS, we performed a GWAS for creatinine eGFR measurement in both people with T2D and people who have not been diagnosed with T1D or T2D. Furthermore, we used the summary statistics released by the SUMMIT consortium [8, 17] to perform a DKD meta-analysis study. While our study confirms the previously published findings in both CKD and creatinine eGFR in T2D, it also uncovers a novel association between NID1 and DKD.

## Methods

### DKD phenotype definition and study population

Clinically, a patient will be diagnosed with CKD if either reduced eGFR (GFR < 60 ml/min/1.73 m^2^) or increased albuminuria defined by urine albumin–creatinine ratio (ACR) ≥ 30 mg/g is present for more than 3 month [19].

These diagnostic criteria do not differentiate DKD from CKD unrelated to T2D. Despite being rarely performed in practice, a kidney biopsy is the gold standard for determining the cause of CKD [9, 20]. These factors make it challenging to define the phenotype of DKD, but considering the timing of a CKD diagnosis relative to the T2D diagnosis can help identify DKD. As described below, we adopted a CKD in T2D vs long duration T2D without CKD definition in this study leading to a diabetes duration that is significantly (*P* = 2.20E−16) shorter in DKD cases compared to complication-free T2D controls (Table 1).

**Table 1:** DKD GWAS sample characteristics

	Control	Case	*P* value
	(n = 7982)	(n = 5141)	
*Characteristics*			
Age (years)	73.8 ± 6.7	76.3 ± 5.6	2.20E−16
Women (%)	37.8	37.4	0.57
Diabetes duration (years)	14.2 ± 3.4	12.5 ± 5.4	2.20E−16
BMI	31.8 ± 5.9	32.6 ± 5.9	1.10E−14
HbA1c	6.7 ± 1.2	6.9 ± 1.4	3.86E−10
Biomarkers*			
eGFR (mL/min/1.73 m^2^)	83.8 ± 10	63.8 ± 18	2.20E−16
Serum creatinine (mg/dl)	0.79 ± 015	1.1 ± 0.6	2.20E−16
Spot urinary creatinine (mg/dl)	113 ± 69	118 ± 73	2.98E−05
Urinary albumin (mg/l)	14.7 (10–1160)	24 (12–6747)	2.20E−16
Urinary sodium (mmol/l)	85 ± 45	78 ± 41	2.20E−16
Urinary potassium (mmol/l)	66.5 ± 33	63 ± 31	6.55E−11

A total of 13,123 individuals with T2D were included in the analysis

Characteristics and biomarkers are reported as the mean ± SD except for women (percentage) and urinary albumin (median and IQR)

*Reported biomarkers values were collected at the first assessment visit to the UKBB for all study participants and before the DKD diagnosis in 84.5% of cases

We used the International Classification of Diseases 10th revision (ICD-10) to identify the study’s cohort from the UKBB. We selected participants with any T2D ICD-10 code (E11.0, E11.1, E11.2, E11.3, E11.4, E11.5, E11.6, E11.7, E11.8, E11.9). Out of 46,308 participants with T2D, we included 13,123 (7982 controls and 5141 cases) unrelated White Europeans in our GWAS. Table 1 summarizes the characteristics of the study’s cohort.

We considered three criteria to define DKD: (1) participants who were assigned the ICD-10 code E11.2 (type 2 diabetes mellitus with kidney complications), (2) participants who were assigned any CKD ICD-10 code (N18.0, N18.1, N18.2, N18.3, N18.4, N18.5, N18.8, N18.9) after being diagnosed with T2D, and (3) participants with GFR < 60 ml/min/1.73 m^2^ and or ACR ≥ 30 mg/g after being diagnosed with T2D. Participants with T2D for more than 10 years and who have not developed any diabetes-related complications were included as controls.

### eGFR calculation

eGFR was calculated using the Chronic Kidney Disease Epidemiology Collaboration (CKD-EPI) creatinine equation:

$$\begin{aligned}{\text{eGFRcr}} &= 142 \times \min \left( {{\text{Scr}}/\upkappa ,1} \right)\alpha \times \max \left( {{\text{Scr}}/\upkappa ,1} \right) \\ &\quad- 1.200 \times 0.9938\;{\text{Age}} \times 1.012\left[ {\text{if female}} \right] \end{aligned}$$

where Scr = serum creatinine in mg/dL, κ = 0.7 (females) or 0.9 (males), α =  − 0.241 (female) or − 0.302 (male), min(Scr/κ, 1) is the minimum of Scr/κ or 1.0, max(Scr/κ, 1) is the maximum of Scr/κ or 1.0, and age (years) [21].

### GWAS quality control

SNPs and individuals with genotyping rate < 98% were excluded. SNPs with minor allele frequency < 5% were excluded. SNPs deviating from Hardy–Weinberg equilibrium test (*P* value< 1E−10 in cases and < 1E−6 in controls) were removed. Individuals who deviate > 3 SD from the samples mean heterozygosity rate were removed. Principal component analysis (PCA) was performed to correct for population substructure. Only White European participants were selected for further analysis. The quality control steps were performed using Plink V 1.9 [22]. The PCA for all studies was performed using bigsnpr [23]. Related individuals were identified using KING [24], and individuals with kinship coefficient ≥ 0.17 were excluded from the analysis.

### Statistical analysis

Genome-wide association analyses were performed using an additive model. The DKD GWAS was corrected for age, sex, duration of diabetes, and the top 10 principal components. The same covariates were adjusted for in the eGFR GWA studies except for the duration of diabetes. The analyses were conducted using Plink V 1.9 [22].

### DKD meta-analysis

We conducted an inverse-variance fixed effects meta-analysis including the summary statistics from our DKD GWAS and the above-mentioned SUMMIT study. The meta-analysis included a total of 18,840 (11,327 cases and 7513 controls) European individuals with T2D; 5717 (3345 cases and 2372 controls) from the SUMMIT study and 13,123 (5141 cases and 7982 controls) from our DKD GWAS. The meta-analysis was performed in Plink V 1.9 [22] combining the effect sizes from both studies. The meta-analysis results included only variants present in both summary statistics reports.

## Results

### Heritability estimation

Heritability (h2) was estimated using the LDAK model [25] from 241,883 directly genotyped SNPs. For DKD, h2 = 0.027 with standard deviation (SD) = 0.03. For eGFR in non-diabetic individuals, h2 = 0.036 with SD = 0.001. For eGFR in T2D, h2 = 0.1 with SD = 0.01.

### DKD GWAS

**Fig. 1:**
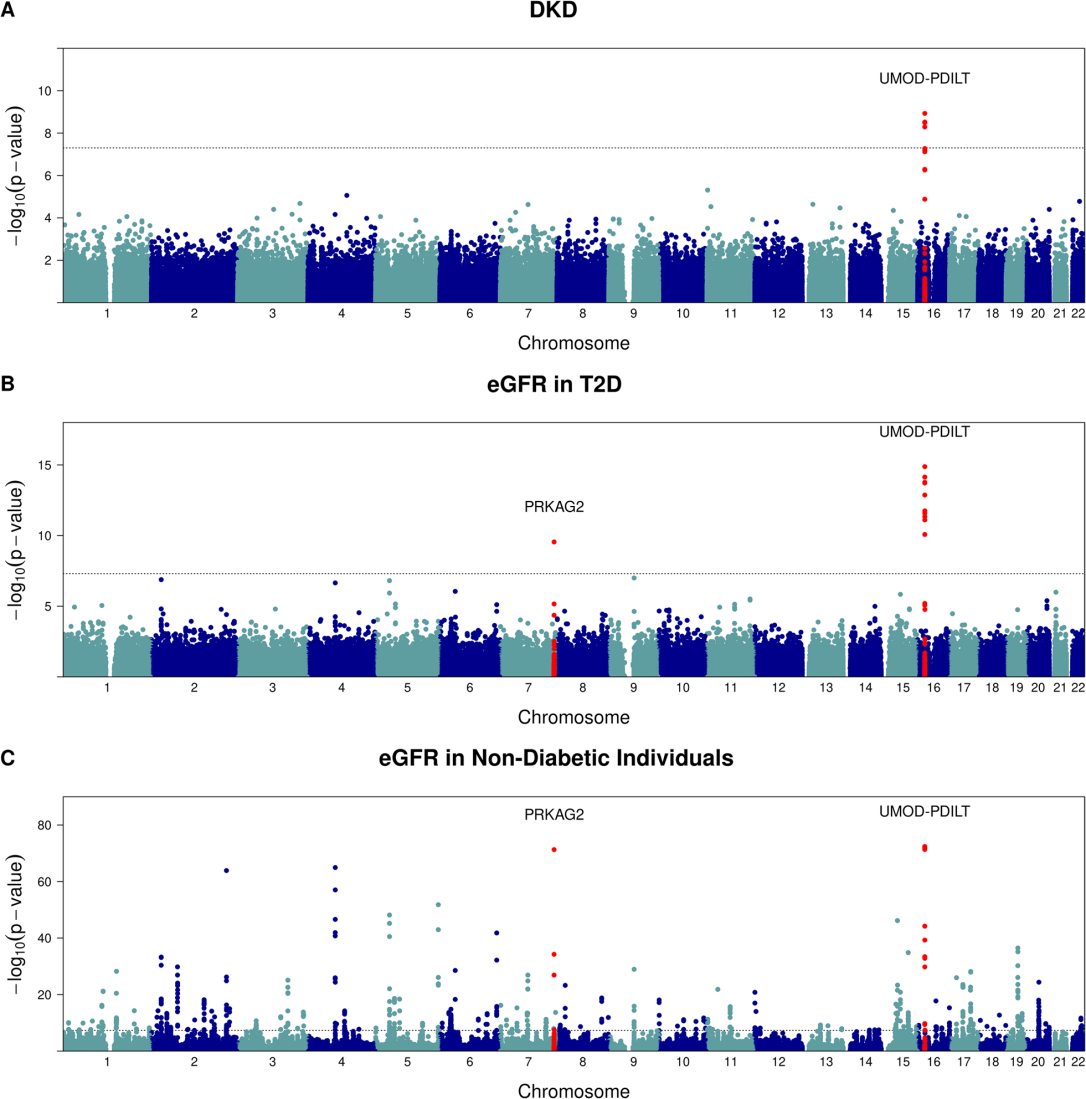
GWAS plots showing **a** diabetic kidney disease (DKD). **b** eGFR measurement in T2D. **c** eGFR measurement in non-diabetic (no T1D or T2D) individuals. The dotted line indicates the genome-wide significance threshold at *P* = 5 × 10^−8^

In the DKD GWAS, only one locus reached genome-wide significance: UMOD-PDILT (Uromodulin-Protein disulfide-isomerase-like protein of the testis) which is located on chromosome 16 (Table 2; Fig. 1a). The same locus was previously reported to be associated with CKD and reduced renal function represented by both serum creatinine and cystatin c eGFR in diabetes [26, 27]. Genomic inflation factor was estimated based on median chi-square statistics, and *λ*_*GC*_ was 1.0 (See QQ plot; Additional file 1: Fig. S1A).

**Table 2:** Genome-wide significant associations with eGFR, DKD, and DKD meta-analysis

								*P* value			OR (95% CI)		Beta (95% CI)	
SNP	CHR	BP	Gene	A1	A2	MAF	DKD	eGFR (T2D)	eGFR (non-diabetic)	DKD Meta-analysis	DKD	DKD Meta-analysis	eGFR (T2D)	eGFR (non-diabetic)
rs12917707	16	20,367,690	UMOD	T	G	0.18	1.17E−09	1.31E−15	1.54E−72	4.45E−08	0.80 (0.75, 0.86)	0.8566 (0.81, 0.9)	1.22 (0.9, 1.5)	0.77 *(0.7, 0.84)*
rs13333226	16	20,365,654	UMOD	G	A	0.19	3.27E−09	7.29E−15	8.96E−73	NS	0.81 (0.75, 0.87)	–	1.18 (0.88, 1.47)	0.77 *(0.7, 0.84)*
rs4293393	16	20,364,588	UMOD	G	A	0.18	5.15E−09	1.71E−14	3.96E−73	NS	0.81 (0.76, 0.87)	–	1.16 (0.86, 1.45)	0.77 *(0.7, 0.84)*
rs7185940	16	20,410,488	PDILT	A	G	0.28	5.36E−08	1.79E−12	1.45E−33	NS	0.84 (0.8, 0.9)	–	0.92 (0.66, 1.71)	0.44 *(0.38, 0.5)*
rs7192797	16	20,411,763	PDILT	A	G	0.28	7.66E−08	4.58E−12	1.10E−33	NS	0.85 (0.8, 0.9)	–	0.9 (0.64, 1.1)	0.44 *(0.38, 0.5)*
rs9926580	16	20,410,547	PDILT	T	C	0.28	6.61E−08	7.68E−12	3.86E−34	NS	0.85 (0.8, 0.9)	–	0.89 (0.63, 1.1)	0.45 *(0.39, 0.5)*
rs11864909	16	20,400,839	PDILT	T	C	0.29	NS	1.35E−13	6.12E−45	NS	–	–	0.95 (0.7, 1.2)	0.51 *(0.45, 0.47)*
rs35208507	16	20,388,929	PDILT	G	A	0.31	NS	2.65E−12	5.26E−40	NS	–	–	0.88 (0.63, 1.13)	0.88 *(0.41, 0.52)*
rs4321205	16	20,375,536	PDILT	T	C	0.38	NS	8.23E−11	1.65E−30	NS	–	–	0.78 (0.54, 1)	0.39 *(0.33, 0.44)*
rs7805747	7	151,407,801	PRKAG2	A	G	0.27	NS	2.79E−10	5.07E−72	NS	–	–	− 0.83 (− 1.09, − 0.57)	− 0.66 (− 0.72, − 0.61)
rs72763500	1	236,116,561	NID1	C	T		NS	NS	NS	2.58E−08	–	0.7933 (0.73, 0.85)	–	–

Italics indicates that the significant *P* value ≤ 5 × 10^−8^

CHR: Chromosome; BP: SNP position (Build 37); A1: minor allele; A2: reference allele; MAF: minor allele frequency; DKD: diabetic kidney disease; eGFR: estimated glomerular filtration rate; and OR: odd ratio

### eGFR GWA studies

To further investigate the UMOD association with renal function, we performed two eGFR GWA studies in two unrelated White European groups selected from UKBB: one in 26,786 individuals (16,323 males and 10,463 females) with T2D and another 339,080 in individuals (186,027 females and 153,053 males) who have not developed T2D or T1D. Both GWA studies were corrected for sex and age at the time of creatinine measurement.

The calculation of eGFR is detailed in the methods section. Genomic inflation factors were calculated based on median chi-square statistics, and *λ*_*GC*_ values were 1.02, and 1.4 for eGFR in T2D GWAS, and eGFR in non-diabetic individuals, respectively (See QQ plots in Additional file 1: Fig. S1b and c). We applied genomic control correction to the latter, and the adjusted *P* values are presented in Table 2, the Manhattan plot (Fig. 1c).

In the T2D eGFR GWAS, 2 loci reached genome-wide significance: UMOD-PDILT and PRKAG2 (Table 2; Fig. 1b). These two loci were previously identified to be associated with CKD, creatinine eGFR, and cystatin c eGFR [27]. The UMOD-PDILT locus was reported in association with creatinine eGFR in T2D [8] and both T1D and T2D combined [8, 27, 28]. The PRKAG2 loci were identified in association with creatinine eGFR in T2D [8].

The same two loci, among many, showed genome-wide significance in the non-diabetic eGFR GWAS (Table 2; Fig. 1c). The difference in the number of the significant signals in the eGFR T2D vs non-diabetic GWA studies could be attributed to the much larger sample size of the non-diabetic GWAS.

The UMOD-PDILT loci have been reported to be associated with the rapid decline in eGFR [29] which could explain its significant association with both eGFR and DKD across all performed GWA studies.

### DKD meta-analysis

As mentioned previously, the SUMMIT consortium released summary statistics for a meta-analysis of seven DKD phenotypes. The overall DKD was defined by microalbuminuria, late DKD (albumin excretion rate ≥ 200 µg/min), or ESRD (eGFR < 15 mL/min/1.73 m^2^ or kidney dialysis or transplant). The study comprised a total of 3345 cases and 2372 controls of European descendent and estimated the heritability of DKD in T2D to be 8%. We chose to include the SUMMIT consortium summary data in our meta-analysis because it is one of the largest DKD GWA studies to date. In addition, it matches both our study’s phenotype definition and population ancestry.

**Fig. 2:**
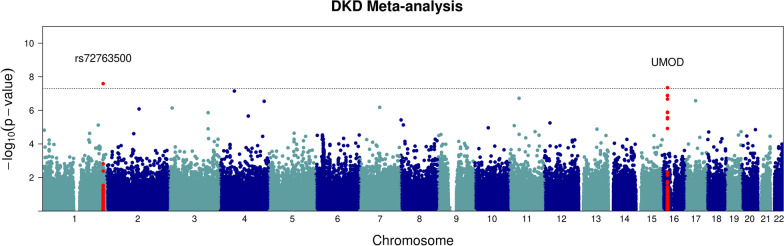
GWAS plot showing: diabetic kidney disease (DKD) meta-analysis. The dotted line indicates the genome-wide significance threshold at *P* = 5 × 10^−8^

The UMOD locus remained significant in the meta-analysis as in the other three GWA studies. We also identified a novel variant, rs72763500 (chr1:236116561), that have not been reported to be associated with DKD before (Table 2; Fig. 2). This novel variant is unique to DKD and did not show a significant association with the eGFR measurement. We queried the NHGRI GWAS catalog (www.genome.gov) to investigate the association of variant rs72763500 with related phenotypes, but it has not been reported by any study. The variant is reported by the Genotype-Tissue Expression (GTEx) project (https://gtexportal.org/home/snp/rs72763500) as a splicing quantitative trait locus (sQTL) of NID1 gene.

We used the Functional Mapping and Annotation of GWA Studies (FUMA GWAS) platform [30] for post-GWAS annotation of our results. To identify the enriched differentially expressed genes (DEG) in the provided GWAS summary statistics, FUMA employs two-sided *t* tests per gene per tissue against all other tissues. Although the tissue enrichment analysis using the GTEx v8 dataset showed no significant (*P*-value ≤ 0.05 after Bonferroni correction) DEG sets, it showed that kidney medulla is the tissue with highest enrichment in our data (Additional file 1: Fig. S2).

Additional file 1: Fig. S3 shows the genes in our data that have been reported by the GWAS Catalog. The related traits to our data are decline in kidney function traits and chronic kidney disease.

## Discussion

For two decades, GWA studies have contributed to our understanding of the biological and physiological aspects of many human traits and diseases. They are powerful tools that have the potential to decipher the pathophysiology of many diseases as well as uncover new drug targets.

DKD is uncurable and usually leads to a debilitating ESRD which greatly reduces the patients’ quality of life. As we mentioned earlier, the genetics of DKD have not yet been extensively studied and, to date, the DKD GWA studies are still limited by the small sample sizes. While moderately extending the sample size of available studies, our study confirms the previously reported variants to be associated with CKD, eGFR in T2D, and reduced renal function. It also reports a novel association between NID1 gene that may be specific to DKD.

NID1 is a sulfated glycoprotein ubiquitous basement membrane protein and is involved in the glomerular basement membrane (GBM) development. We queried the Human Protein Atlas single-cell RNA expression database (https://www.proteinatlas.org/ENSG00000116962-NID1/single+cell+type) for NID1 tissue enrichment. NID1 is highly expressed in mesenchymal cells in adipose tissue, breast, esophagus, endometrium, liver, skeletal, and heart muscles. In the kidney, it is more abundant in the proximal tubular cells of the nephron in comparison to the distal tubular cells and collecting duct cells. On the contrary, UMOD is more abundant in the distal tubular cells and collecting duct cells compared to the proximal tubular cells.

The GBM is mainly composed of laminin, type IV collagen, nidogens (NID1 and NID2), and heparan sulfate proteoglycan [31, 32]. NID1 binds both laminin and type IV collagen [33]. Deletion of one of the nidogens in mice does not affect the development of basement membranes, but deletion of both genes leads to basement membrane abnormalities in the lungs and heart and subsequently perinatal death. The nidogen double mutant mice developed normal glomerular basement membrane, but the tubular basement membrane was sometimes absent or altered [34]. The effect of nidogen mutation on the human kidney is still largely unknown.

Our meta-analysis finding, although biologically relevant, is limited by the lack of a replication study. More studies are needed to substantiate and further investigate the role of NID1 in human DKD.

## Supplementary Information

**Additional file 1: Figure S1**. Q–Q plots: (A) Diabetic Kidney Disease (DKD). (B) eGFR measurement in T2D. (C) eGFR measurement in non-diabetic (no T1D or T2D) individuals. λ = genomic inflation factor. **Figure S2** Tissue enrichment analysis of the diabetic kidney disease (DKD) meta-analysis showing the levels of both up-regulated and down-regulated differentially expressed genes (DEG) in various tissues. Significant enrichment level is Bonferroni corrected *P*-value ≤ 0.05.** Figure S3** GWAS Catalog reported genes: showing genes, from the diabetic kidney disease (DKD) meta-analysis, reported by the GWAS Catalog.

## Data Availability

The datasets generated during and/or analyzed during the current study are available from the corresponding author upon reasonable request.

## Abbreviations

CKD: Chronic kidney disease
DKD: Diabetic kidney disease
eGFR: Estimated glomerular filtration rate
ESKD: End-stage kidney disease
FUMA GWAS: Functional mapping and annotation of genome-wide association studies
GTEx: Genotype-tissue expression
GWA: Genome-wide association
GWAS: Genome-wide association study
NID1: Nidogen-1
PCA: Principal component analysis
SNP: Single nucleotide polymorphism
sQTL: Splicing quantitative trait locus
SUMMIT: Surrogate markers for micro- and macro-vascular hard endpoints for innovative diabetes tools.
T1D: Type 1 diabetes
T2D: Type 2 diabetes
UKBB: UK Biobank
