# Supplementary material for: Nidogen-1 could play a role in diabetic kidney disease development in type 2 diabetes: a genome-wide association meta-analysis
Source: Hum Genomics. 2022 Oct 21;16:47. doi: 10.1186/s40246-022-00422-y (PMC9587571; doi:10.1186/s40246-022-00422-y)
Supplement: Supplementary file 1 — Additional file 1: Figure S1. Q–Q plots: (A) Diabetic Kidney Disease (DKD). (B) eGFR measurement in T2D. (C) eGFR measurement in non-diabetic (no T1D or T2D) individuals. λ = genomic inflation factor. Figure S2 Tissue enrichment analysis of the diabetic kidney disease (DKD) meta-analysis showing the levels of both up-regulated and down-regulated differentially expressed genes (DEG) in various tissues. Significant enrichment level is Bonferroni corrected P-value ≤ 0.05. Figure S3 GWAS Catalog reported genes: showing genes, from the diabetic kidney disease (DKD) meta-analysis, reported by the GWAS Catalog. [file 40246_2022_422_MOESM1_ESM.docx]

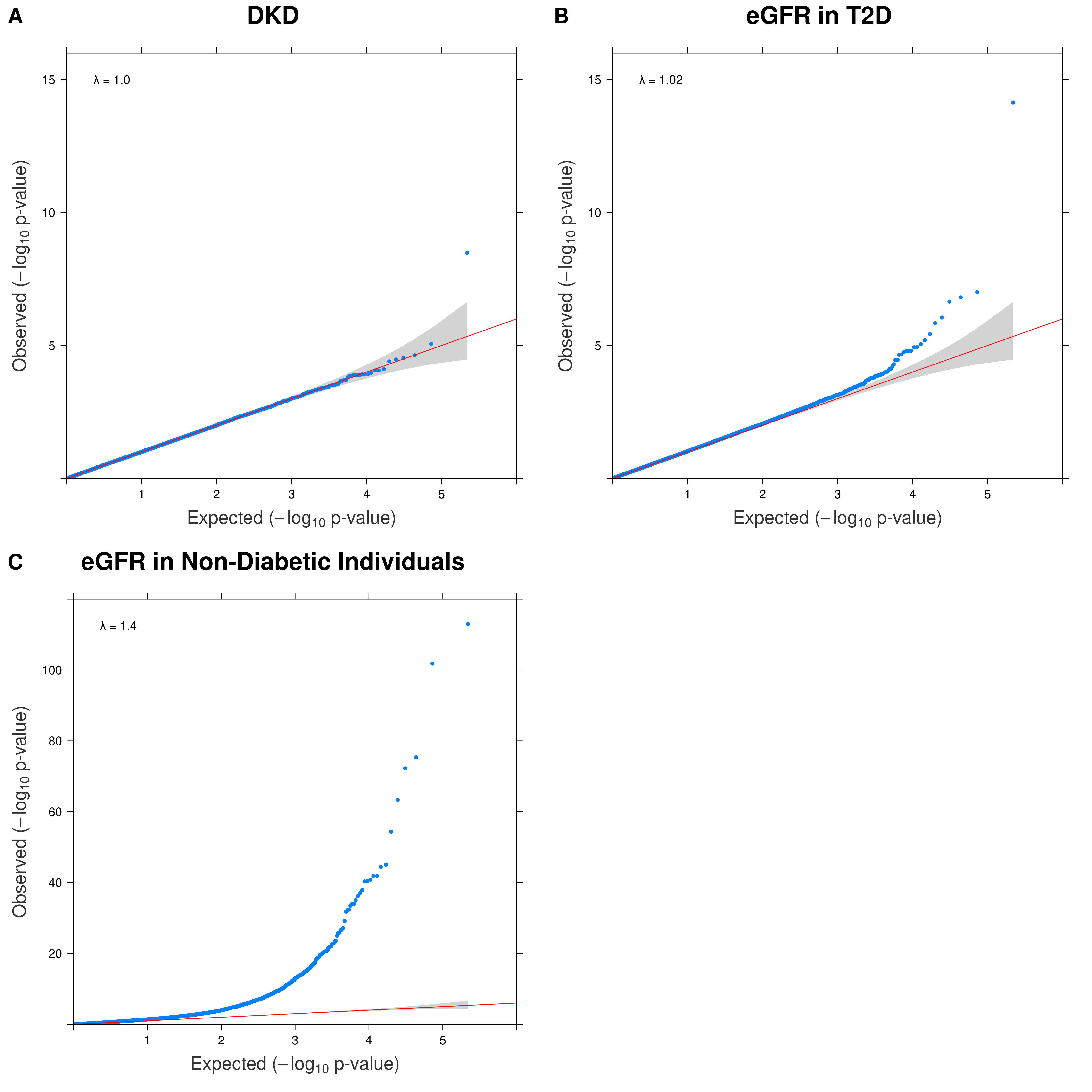


**Supplementary Figure 1.** Q-Q plots: (**A**) Diabetic Kidney Disease (DKD). (**B**) eGFR measurement in T2D. (**C**) eGFR measurement in non-diabetic (no T1D or T2D) individuals. λ = genomic inflation factor.


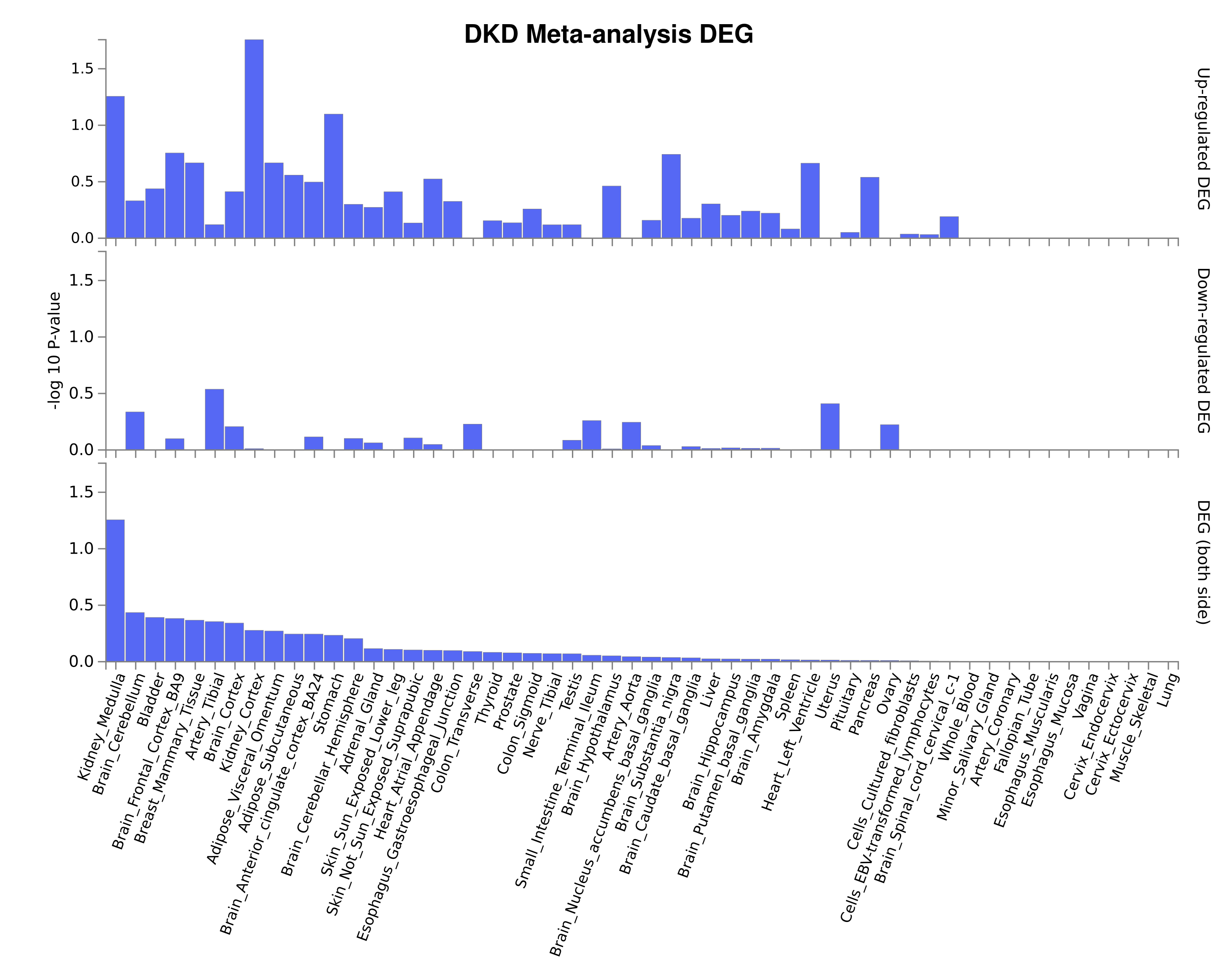


**Supplementary Figure 2.** Tissue enrichment analysis of the diabetic kidney disease (DKD) meta-analysis showing the levels of both up-regulated and down-regulated differentially expressed genes (DEG) in various tissues. Significant enrichment level is Bonferroni corrected P-value ≤ 0.05.


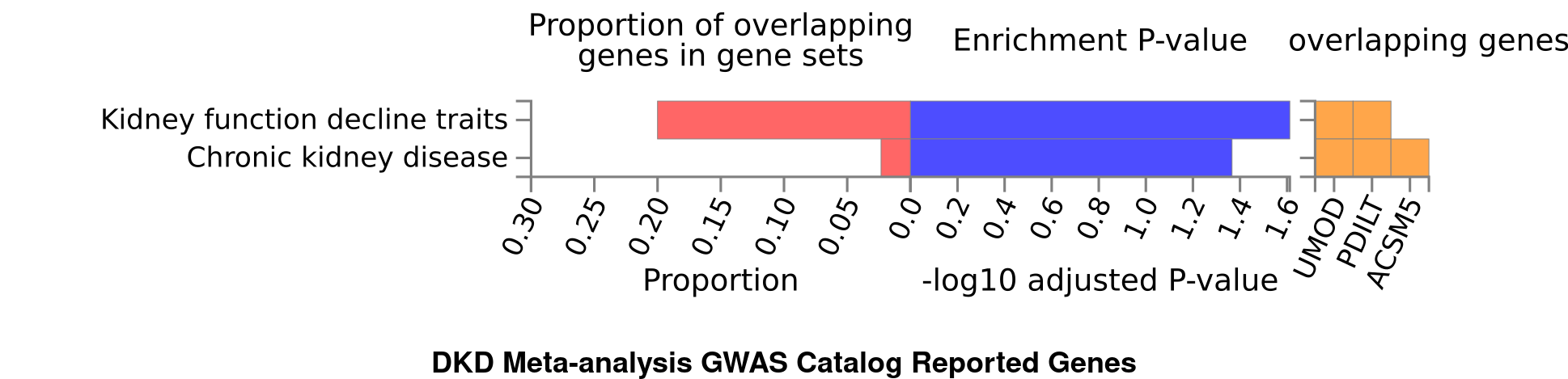


**Supplementary Figure 3.** GWAS Catalog reported genes: showing genes, from the diabetic kidney disease (DKD) meta-analysis, reported by the GWAS Catalog.
